# Retinal Artery Occlusion: A Review of Current Management Practices

**DOI:** 10.18502/jovr.v19i4.16559

**Published:** 2024-12-31

**Authors:** Hannah J. Yu, Sophia Choi, Rodney Guiseppi, Touka Banaee

**Affiliations:** ^1^John Sealy School of Medicine, University of Texas Medical Branch, Galveston, Texas, USA; ^2^Department of Ophthalmology and Visual Sciences, University of Texas. Medical Branch, Galveston, Texas, USA; ^4^Hannah J. Yu: https://orcid.org/0000-0003-3827-4915

**Keywords:** Branch Retinal Artery Occlusion, Central Retinal Artery Occlusion, Management, Retinal Artery Occlusion, Systematic Review, Treatment

## Abstract

Retinal artery occlusion (RAO) is a well-characterized ischemic ophthalmic event that may result in sudden and devastating vision loss. The etiology of RAO may vary including both arteritic and non-arteritic causes and the location of the lesion can extend from the ophthalmic artery to the branches of the central retinal artery. Given this variable causes of RAO, the clinical presentation and extent of vision loss may also differ from case to case, necessitating a prompt and thorough evaluation, including a full stroke work up. While there is currently no widely accepted standard for the treatment of RAO, there are several proposed methods that have been or are currently being investigated through retrospective studies and prospective trials. The current article aims to provide a review of the pathophysiology, clinical presentation, and management of RAO in addition to presenting a systematic review of recently published studies on treatment options for RAO.

##  INTRODUCTION

Retinal artery occlusions (RAOs) are an ophthalmic emergency and may result in sudden and severe vision loss. RAOs may be caused by an embolus or thrombus, vasculitis, trauma, or vasospasm of the central retinal artery (CRAO) or a branch of the central artery (BRAO), and result in widespread ischemia of the retinal tissue.^[1]^ Although some patients may regain some visual acuity (VA) after RAO, the vast majority experience extensive vision loss with poor prognosis and very few options for management.^[2,3]^

The management and treatment of RAO and retinal ischemia in general is still widely debated among clinicians today. While it is widely accepted that RAO requires emergency management and extensive further work up for underlying etiology, there is no accepted, standard-of-care treatment protocol for patients presenting with non-arteritic RAO. As retinal ischemia can be the result of a wide range of pathologies, a thorough exam and work up for these differentials must be performed promptly. Additionally, multiple different treatment modalities have been studied, ranging from ocular massage to thrombolysis and surgical therapy, though larger-scale trials are still needed to demonstrate true improvement in patient outcomes without significant adverse events.

The current article aims to describe the clinical presentation, pathophysiology, diagnosis, and management of acute RAO in addition to presenting a systematic review of recent studies in the various treatment modalities of acute, non-arteritic RAO.

##  METHODS

Two electronic databases (PubMed and Scopus) were searched for the current systematic review of recent developments in the management of RAO. A PubMed search was performed with the search terms: (Retinal Artery Occlusion) AND ((Treatment) OR (Management)) limited to articles published between 2019 to 2023. Scopus search was performed similarly with the additional limitations to only search for the “article” document type and English articles.

Articles were further screened by two authors independently for content of titles, abstracts, and full-text articles to ensure inclusion of human studies investigating treatment of non-arteritic RAO with reported visual or functional and anatomic outcomes. Included study types were meta-analyses, prospective studies, retrospective studies, and clinical trials. Articles were excluded if they were solely case reports, systematic reviews, review articles, or case series with an *n*-value ≤ 5. If an article included more than one study type (i.e., retrospective study and meta-analysis) and both studies met inclusion criteria, both parts were included as separate studies. If only one part of the paper met inclusion criteria, only that part of the study was included. Articles were also excluded if they were non-English or preprints.

### Pathophysiology and Etiology

The primary blood supply for the globe is the ophthalmic artery branch of the internal carotid artery. The ophthalmic artery then splits into several branches, including the lacrimal artery, long and short posterior ciliary arteries, and the CRAO. While the ciliary arteries are the primary blood supply for the choroid and the outer retina, the CRAO primarily supplies the inner retina which consists of the nerve fiber layer (NFL), the ganglion cell layer, and the inner plexiform layer.^[1,4]^ Additionally, about one-third of patients may present with a cilioretinal artery branch of the posterior ciliary arteries which can also supply the fovea.^[5]^ Although the optic nerve head is also supplied by the CRAO, anastomotic supply from the choroidal vessels can be sufficient to supply the optic nerve head in the event of CRAO.^[6]^

In RAOs, the CRAO or a branch of this artery is occluded by emboli, thrombi, or vasculitis from a variety of etiologies, thus causing hypoperfusion and ischemia of the inner retina. Although there may be some passive diffusion of oxygen from the outer retina, the retina is very sensitive to ischemia, resulting in irreversible damage after short periods of time without adequate perfusion. Some studies with animal models have suggested that functional retina would be viable if reperfusion occurs in < 1.5 hours;^[7,8]^ however, others have suggested that irreversible damage can occur in as little as 12–15 minutes after occlusion.^[9]^ Although the exact length of time before permanent damage may occur in humans is still unclear, this sensitivity results in poor prognosis for patients who develop RAOs, with < 10–20% of patients experiencing meaningful improvements in VA after initial presentation.^[2,10,11]^

As with most cerebrovascular accidents, the most common cause of RAO is attributed to emboli from the systemic circulation. Other causes include thrombosis or inflammatory processes. In a 2010 study of 439 patients with RAO, 53.3% presented with non-arteritic CRAO, 32.1% with BRAO, and 3.4% with arteritic RAO.^[12]^ Eighty-five percent of patients with non-arteritic CRAO had follow up with cervical vessel imaging, with 71% demonstrating an ipsilateral carotid plaque. Additionally, 56% had echocardiography performed with 52% of that population demonstrating an abnormal result with an embolic source.^[12]^

There are many etiologies of RAO emboli, including calcium, cholesterol, and fibrin-platelet emboli. Cholesterol emboli, sometimes called Hollenhorst plaques, and fibrin-platelet emboli are common causes of RAO and typically originate from carotid plaques.^[13,14]^ Calcium emboli are also common and typically arise from calcified cardiac valves. They can often be differentiated by their appearance with cholesterol emboli appearing orange, fibrin-platelet emboli appearing dull white, and calcium emboli appearing a brighter white.^[13]^ Rarer types of RAO embolism include talc, septic, fat, amniotic fluid, metastatic tumor, and air emboli.^[14]^

The type of emboli in RAO can play a role in the visual prognosis of patients, as some are more susceptible to dislodgement and dissolution. In a study by Cho et al, retrospective analysis of patients with RAO and visible emboli demonstrated that platelet-fibrin emboli were often associated with emboli that migrated peripherally or disappeared during the follow up period.^[15]^ Cholesterol emboli were also shown to migrate. Eighty percent of observed eyes with migrating emboli resulted in early complete reperfusion of the retina. Cholesterol emboli were also shown to be associated with emboli without migration along with calcium emboli, which resulted in late incomplete reperfusion. The reason for decreased migration of some emboli may be due to size and chemical makeup of the emboli, as platelet-fibrin emboli are more likely to be smaller and dissolvable with thrombolytic treatment, while calcium emboli or large atheromatous plaques may not dissolve as easily.^[15]^ Additionally, large emboli may become lodged proximal to the lamina cribrosa resulting in more significant ischemia and more devastating visual loss.^[16]^ These may be visible on transorbital ultrasonography.^[17]^

Thrombotic causes are also a possible etiology of RAO and are largely due to atherosclerosis, inflammatory disease, and hypercoagulable states.^[13]^ In an elderly patient with concomitant jaw claudication and tenderness at the temple, giant cell arteritis (GCA) is an important differential cause of inflammatory thrombosis and must be addressed urgently.^[18]^ GCA is also an important cause of anterior ischemic optic neuropathy and choroidal ischemia, and the presentation is dependent on the primary location of inflammation in the ocular vasculature.

In young patients with low risk of atherosclerotic disease, vasculitis and hypercoagulable work ups are also crucial to prevent further sequelae of undiagnosed disease.^[19]^ Screening of these often require a vast number of tests due to the many etiologies of hypercoagulability.^[20]^ Excess states of certain procoagulant factors such as Factor VIII must be considered as well as deficiencies of anticoagulant factors like antithrombin, protein C, and protein S.^[20]^ Some patients may also be affected by genetic mutations in their coagulation cascade such as Factor V Leiden.^[21]^ Antibody screens for conditions such as antiphospholipid antibody syndrome and systemic lupus erythematosus should also be performed. Hypercoagulability may also be diagnosed through excess blood products like thrombocytosis, leukocytosis, or erythrocytosis.^[20]^

### Clinical Features and Diagnosis

Patients with symptomatic, acute RAO typically present with acute, painless vision loss of varying severity that occurs over seconds.^[22]^ Among patients with CRAO, VA may be as poor as counting fingers, while patients with BRAO may present with partial visual field loss. If the RAO is cilioretinal sparing, patients can also present with little to no change in VA.^[10,23]^ Patients may also report a history of transient vision loss associated with amaurosis fugax.^[13]^

When presenting with sudden onset, monocular vision loss, workup should include a complete ophthalmic exam with dilation to differentiate the cause. While RAO may present with an ipsilateral relative afferent pupillary defect, fundus exam varies depending on the timing of presentation. Classically, patients will present with retinal whitening from retinal edema [Figures 2 & 3A] and opacification of the NFLs, cherry red spot, retinal emboli [Figure 3B], and slow segmental blood flow (“boxcarring”), however, these findings typically develop over multiple hours, and some findings such as edema and retinal emboli can resolve over time.^[22]^ Signs of chronic RAO include atrophy of the inner retina, optic disc pallor, attenuated vessels, and retinal pigment epithelial mottling [Figures 3B & 4B].

### Imaging

#### Fundus photography

Imaging should be utilized to rule out other differentials, especially when fundus exam appears otherwise normal [Figures 2 & 3]. While color fundus photography may be helpful in identifying fundus lesions, it should not replace a complete fundus exam as standard of care. However, color fundus photography can also help rule out other causes of decreased vision such as hemorrhage, detachment, or optic neuropathies.

#### Optical coherence tomography

Optical coherence tomography (OCT) can also be utilized to better elucidate various biomarkers following RAO.^[24]^ During the acute phase of RAO, OCT findings may include intraretinal edema, inner retinal layer hyperreflectivity, inner and outer retinal thickening, and disorganization of the retinal layers structure [Figure 4A].^[25]^ Additionally, one of the first signs of RAO on OCT may be paracentral acute middle maculopathy [Figure 3A], characterized by a hyperreflective band-like lesion at the inner nuclear layer.^[22,26,27]^ Chronically, as edema resolves, the inner retina may atrophy, resulting in overall thinning of the retina [Figures 3B & 4B].

In a study by Mangla et al, these imaging biomarkers were demonstrated across RAO severity as well.^[28]^ In this study, OCTs were reviewed from 39 eyes of patients with acute CRAO as defined by recent history of sudden, painless loss of vision with presence of retinal thickening and whitening, retinal vessel attenuation and a cherry red spot at the macula.^[28]^ Characteristics of OCTs were described, including presence or absence of posterior vitreous opacities, inner and middle retinal features, neurosensory detachment, and prominent retinal pigment epithelium (RPE). Severity of CRAO was then categorized based on these descriptive findings. Presence of middle or inner layer opacification without inner retinal layer thickening or absence of inner retinal stratification was defined as mild CRAO. Moderate was defined as those with middle or inner retinal layer opacification and inner retinal layer thickening but without absence of inner retinal layer stratification. Severe CRAO was defined as any OCT with middle or inner retinal layer opacification, inner retinal layer thickening, and absence of inner retinal layer stratification.^[28]^

Among eyes with mild CRAO, opacification of the middle retinal layers was the most prominent finding along with a prominent middle limiting membrane (p-MLM) present in the first week in 77% of eyes. After one week, there was consistent thinning of the inner retinal layers and persistence of the p-MLM sign. In patients with moderate CRAO, the most common acute OCT findings were inner retinal opacification and increased retinal thickness, with some prominent hyperreflective RPE and a p-MLM sign. Over time, however, inner retinal opacification and thickness reduced with eventual thinning of retinal layers and flattened foveal contour. Among eyes with severe CRAO, patients were all found to acutely have total inner retinal opacification with significant thickening, loss of inner retinal layer differentiation, and prominent RPE hyperreflectivity. Similar to mild and moderate CRAO, though, chronically thinning of retinal layers was seen.^[28]^

Additional OCT findings such as inner retinal fluid, neurosensory detachment, internal limiting membrane (ILM) detachment, inner retinal hyperreflective foci, and posterior vitreous hyperreflective opacities were not observed in any eyes with mild CRAO, but were observed in some eyes with moderate disease and most commonly in eyes with severe CRAO.^[28]^

#### Fluorescein and Optical Coherence Tomography Angiography

Angiography can also be helpful in cases of RAO. Although not necessary for diagnosis, fluorescein angiography may acutely demonstrate delayed filling of the retinal arteries, hyperfluorescent leakage, and delayed arteriovenous transit time and may be useful in identifying the etiology and the extent of retinal ischemia [Figure 5].^[1,22,29]^ Similarly, indocyanine green (ICG) angiography can demonstrate delayed filling of the choroidal vasculature and arteriovenous transit time and can be more helpful in identifying more proximal arterial occlusions like in GCA. Nevertheless, though fluorescein and ICG angiography have multiple uses in acute cases of RAO, they are also time-intensive and more invasive than is necessary for diagnosis, with limited resolution and depth information. Additionally, as time passes, the occlusion may resolve, resulting in vast reperfusion of the retina on angiography, although some small vessel ischemia may still be identifiable.^[30,31]^

OCT angiography (OCT-A), however, may provide a quicker, less invasive option for visualizing retinal blood flow in cases of RAO with higher resolution, albeit without demonstration of vascular permeability [Figure 6]. For patients who may have contraindications to receiving fluorescein injections, such as in the case of allergies or kidney disease, OCT-A provides an excellent alternative to fluorescein angiography for visualization of the retinal vasculature.^[32,33]^ It may even provide more specific and sensitive information regarding blood flow in different layers of the retina.^[34,35,36,37]^ OCT-A may also be more useful in demonstrating small vessel dropout in chronic cases of RAO.^[31,38]^

This detailed characterization of the vascular effects of CRAO has also proved useful in predicting visual effect of RAO. In a 2024 retrospective study of 62 eyes with acute non-arteritic CRAO, OCT-A imaging was used to determine vasculature characteristics of eyes with non-arteritic CRAO which were then correlated with VA.^[39]^ When compared to patients' non-diseased fellow eye, the vessel density in the deep capillary plexus was significantly decreased across eyes with mild, moderate, and severe CRAO. While the choriocapillaris flow was significantly decreased in eyes with moderate to severe CRAO when compared to their fellow eye, the vessel density in the superficial vascular plexus at the fovea was significantly decreased in eyes with mild CRAO when compared with their fellow eye and when compared with eyes with moderate to severe CRAO. The authors postulated that these results indicate that different vessels may be affected in the acute stage depending on the severity of the occlusion, with mild CRAO affecting superficial vessels and severe CRAO affecting deeper vessels.

Regarding clinical significance, this study also found that increased vessel density of the superficial vascular plexus at the fovea and the nasal parafovea was correlated with worse VA, hypothesized to be due to increased vessel density compensating for more severe retinal injury.^[39]^ This finding was consistent with previous findings by Lu et al.^[40]^

A caveat to the use of OCT-A versus fluorescein, however, is that OCT-A may be unable to detect vessels with slow flow, thus making it appear as though those vessels are completely obstructed.^[30]^ In a case reported by Bonnin et al, a patient with BRAO was imaged with both OCT-A and fluorescein angiography early in the encounter and 6 hours later.^[41]^ In the first set of images, slow perfusion was present in the occluded branch on fluorescein angiogram, with no detection of that vessel on OCT-A. In the set of images taken 6 hours later, flow was detected in both types of images.

### Differential Diagnoses

Differential diagnoses for RAO include ophthalmic artery occlusion (OAO), ocular ischemic syndrome (OIS), and carbon monoxide poisoning. As indicated by the name, OAO is caused by occlusion of the ophthalmic artery which supplies the inner and outer layers of the retina.^[22]^ As such, vision loss in OAO is generally more severe than CRAO at hand motion or worse. Additionally, as the choroid is supplied by the ophthalmic artery, in OAO, on exam, no cherry red spot would be observed.

OIS is a hypoperfusion condition usually caused by carotid artery occlusion and may also present with significant vision loss.^[42]^ However, OIS typically also presents with ocular pain which is not seen in RAO. Fundus exam also differs from RAO with microaneurysms, cotton wool spots, retinal hemorrhages, and dilated retinal veins, resulting in OIS being more commonly confused with retinal vein occlusion or diabetic retinopathy.^[43]^ Similarly, although visual dysfunction from carbon monoxide poisoning may present with a similar cherry red spot on fundus exam, patients will also experience eye swelling, ocular pain, photophobia, and tearing.^[44]^

### Risk Factors

Risk factors for RAO can vary greatly due to the various etiologies of occlusion, although risk factors are largely similar to those of cerebrovascular accidents. These risk factors include any inflammatory or hypercoagulable state, age, hypertension,^[45]^ diabetes,^[46]^ smoking,^[47]^ estrogen therapy,^[48]^ and atrial fibrillation.^[49,50,51,52]^ Additionally, studies have shown that when controlling for confounding comorbidities, patients with end-stage renal disease also have nearly a threefold increased risk for RAO compared to controls.^[53,54]^ Patients with autoimmune conditions^[55,56,57,58,59,60]^ and vasculitides^[61,62,63,64,65]^ are also at a higher risk for RAO.

Carotid artery stenosis is another important factor in predicting risk of RAO. In a 1988 study by Merchut et al, 34 patients with symptomatic RAO without obvious clinical etiology of the RAO were assessed for ipsilateral carotid artery disease.^[66]^ Of these, 23% had a total occlusion of their internal carotid, 12% had a stenosis > 80%, 15% had stenosis < 60%, and 35% had a plaque/ulcer. Only 15% of patients demonstrated normal internal carotid artery on angiogram. More recently, in the randomized controlled EAGLE trial of patients with non-arteritic RAO, a sub-analysis of vascular risk factors and underlying diseases were assessed and it was found that 40% of patients had a carotid artery stenosis of at least 70%.^[67]^ Additionally, a study of RAO patients who received diffusion-weighted magnetic resonance imaging (MRI) demonstrated that patients with carotid stenosis or cardioembolic sources of their RAO were more likely to have CRAO (38.9%) versus BRAO (6.7%).^[68]^

RAOs may also result as a rare complication of anti-vascular endothelial growth factor (VEGF) injections. Several studies have reported rates of RAO following anti-VEGF injections for retinal vascular disease, the first of which was published by the International Intravitreal Bevacizumab Safety Survey.^[69]^ This study reported only one case of CRAO after 7113 injections of bevacizumab. In the years since, multiple case series and larger-scale studies have demonstrated RAO after intravitreal anti-VEGF injections varying in severity from transient occlusion to severe vision loss.^[70,71,72]^ It has been theorized that these cases of RAO may be related to the vasoconstrictive properties of anti-VEGF therapy.^[73]^ In 2010, Papadopoulou et al published a case series of 11 patients being treated for neovascular age-related macular degeneration who demonstrated significant retinal arterial vasoconstriction after multiple intravitreal ranibizumab injections.^[74]^ Similar results were shown by Soliman et al among patients with diabetic macular edema treated with bevacizumab.^[75]^

**Figure 1:**
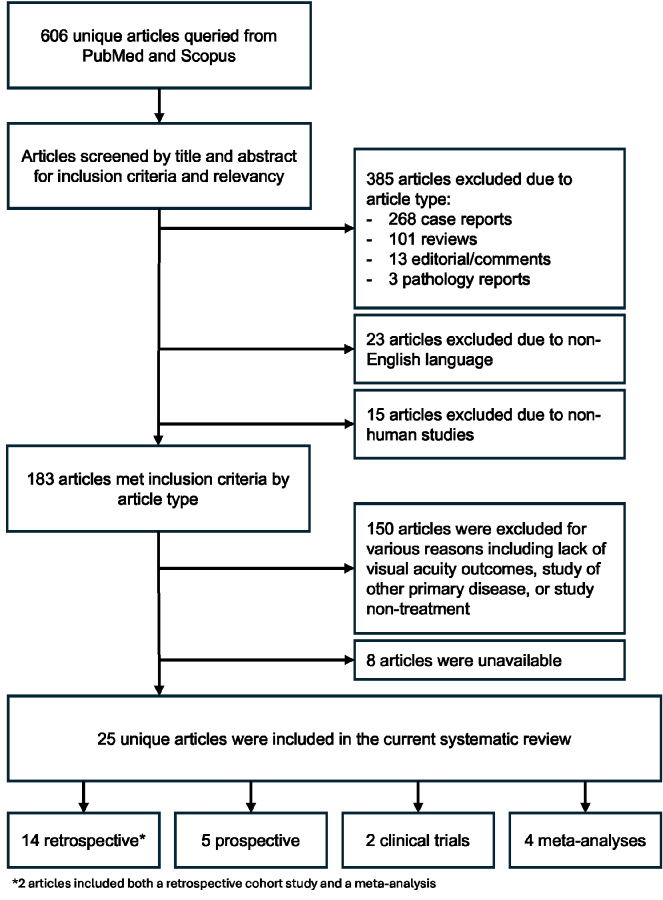
Flow chart of article selection. PubMed and Scopus were searched for articles and screened by two authors independently for article type and content with 25 articles included in the final review.

**Figure 2:**
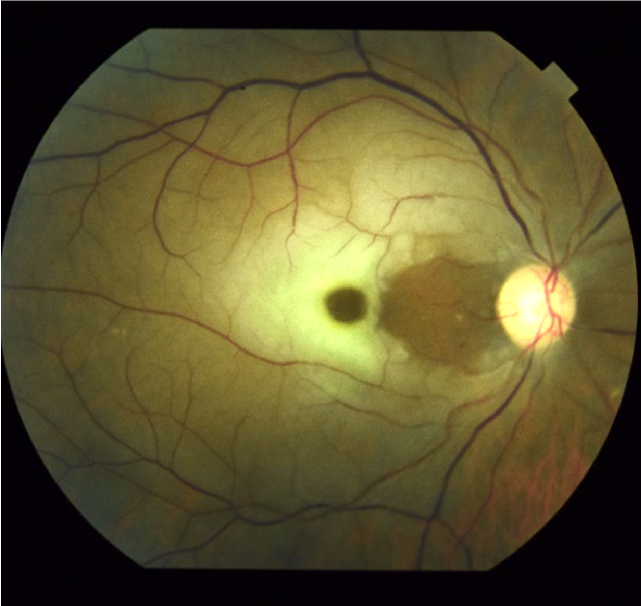
Fundus photo of acute central retinal artery occlusion with cilioretinal artery sparing. The area nourished by the cilioretinal artery has retained its normal color with the rest of the retina showing retinal whitening. A prominent cherry red spot is visible.

**Figure 3:**
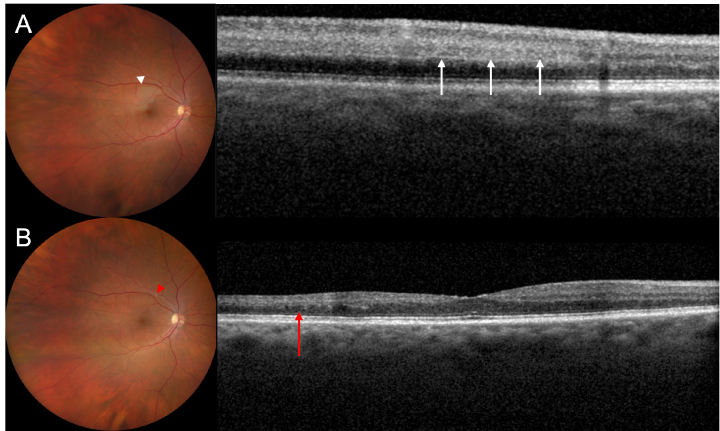
Case of branch retinal artery occlusion (BRAO). (A) The acute phase demonstrates retinal whitening (white arrowhead), parafoveal acute middle maculopathy characterized by hyperreflectivity of the middle retinal layers (white arrows), and retinal thickening. (B) After resolution of the acute changes, the embolus causing the RAO is visible in the fundus image (red arrowhead) along with retinal atrophy (red arrow).

**Figure 4:**
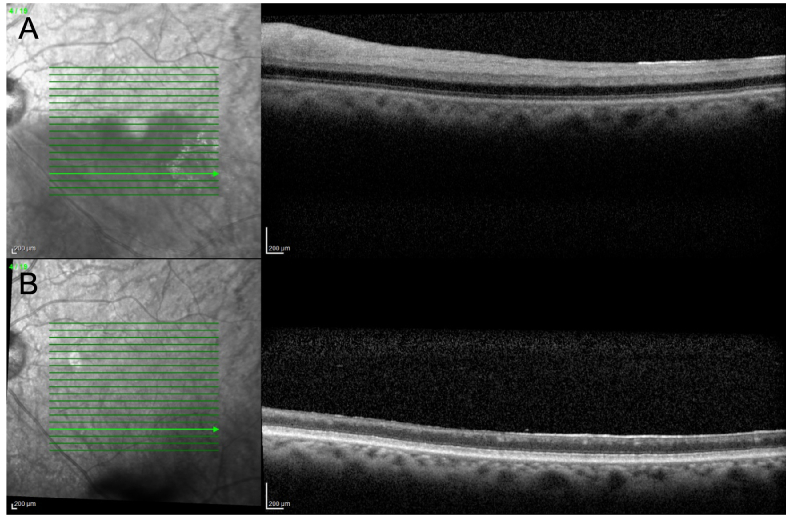
Optical coherence tomography of an acute branch retinal artery occlusion. (A) Acute stages demonstrate hyperreflective inner retinal layers with preservation of the outer retinal structure. (B) Chronic stages demonstrate atrophy of the inner retinal layers with preserved outer retina.

**Figure 5:**
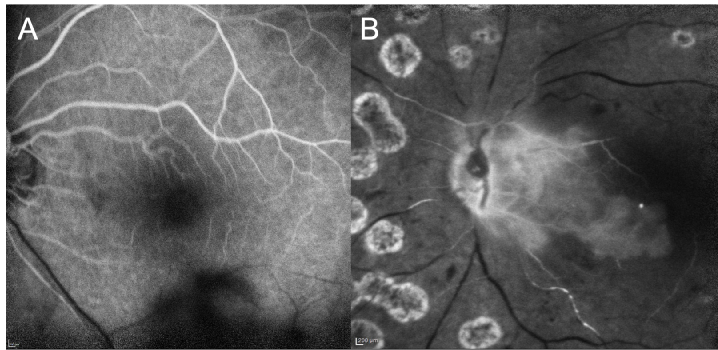
Fluorescein angiography (FA) of eyes with retinal artery occlusion (RAO). (A) In an eye with branch RAO, FA shows delayed filling of the narrowed inferior retinal artery in comparison to the superior retinal arteries with absence of fluorescein in the inferior retinal veins. (B) In an eye with central RAO and preserved cilioretinal artery circulation, proximal retinal arterial walls have been stained but without flow in the retinal arteries in contrast to the area supplied by the cilioretinal artery.

**Figure 6:**
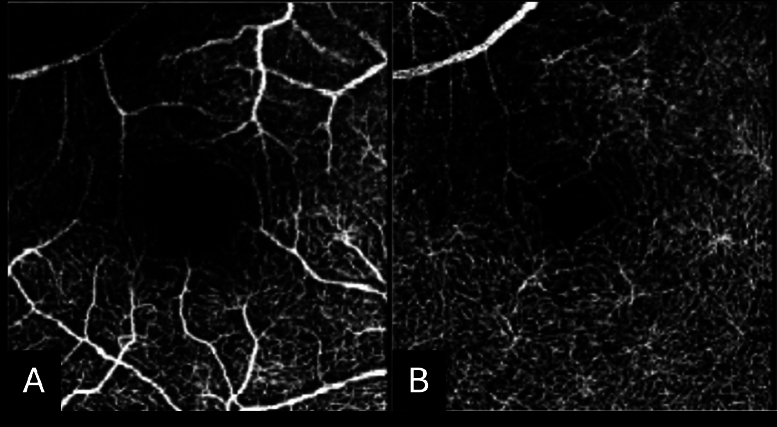
Optical coherence tomography angiography (OCTA) of a patient with a branch retinal artery occlusion. OCTA of the (A) superficial capillary network and the (B) deep capillary network demonstrate decreased flow supertemporally to the fovea.

**Table 1:** Included prospective and retrospective thrombolytic studies.


**Author ${\;}^{Ref}$ **	**Study type**	***N*** **, Cohort**	**Intervention**	**Visual acuity outcomes**
Kim J *et al* ^[96]^	Prospective	*N *= 38, CRAO (Ophthalmic artery lesion) *N* = 62, CRAO (Carotid artery lesion)	Super-selective IAT (urokinase) with DSA	Clinically significant improvement in VA in > 10%
Ko SJ *et al* ^[95]^	Retrospective	*N* = 44, CRAO	IAT (urokinase)	All patients: VA improvement from 1.65 to 1.18 logMAR (*P* = 0.114) Incomplete RAO: 0.08 to 0.06 logMAR (*P* = 0.933) Subtotal RAO: 1.81 to 1.36 logMAR (*P* = 0.014) Total CRAO: 2.36 to 2.42 logMAR (*P* = 0.642)
Sobol EK *et al* ^[93]^	Retrospective	*N* = 15, CRAO (Sx < 12 hr)	IAT (tPA, transfemoral)	VA from 2.18 to 1.42 logMAR (*P* = 0.0061)
Kobkitsuksakul C *et al* ^[94]^	Prospective	*N* = 9, CRAO (Sx < 24 hr)	IAT (tPA) and nimodipine	VA improvement of 0.78 logMAR (*P* = 0.001)
Mac Grory B *et al* ^[97]^	Prospective	*N* = 16, RAO (IAT < 4.5 hr) *N* = 8, RAO (IAT 4.5 to 6 hr) *N* = 87, RAO (Untreated)	IVT (alteplase)	VA improvement of 1.0 logMAR (IVT) versus 0.3 logMAR (Untreated, *P* = 0.001)
Baumgartner P *et al* ^[98]^	Retrospective	*N* = 47, RAO (IVT) *N* = 34, RAO (Conservative treatment)	IVT or conservative ${\;}^{a}$	VA improvement of 0.5 logMAR (IVT, *P* < 0.001) versus 0.4 logMAR (conservative, *P* < 0.05)
Raber FP *et al* ^[100]^	Retrospective	*N* = 16, CRAO (IVT < 4.5 hr) *N* = 21, CRAO (conservative > 4.5 hr)	IVT (rtPA) or conservative ${\;}^{a}$	VA improvement from 2.3 to 2.1 logMAR (IVT) versus 2.3 to 2.3 logMAR (conservative, no *P*-value)
Kozner P *et al* ^[99]^	Retrospective	*N* = 16, CRAO (IVT) *N* = 30, CRAO (no treatment)	IVT	VA improvement from 0.001 to 0.050 decimal equivalents (early IVT; *P* = 0.04 compared to all other cohorts)
Schönecker S *et al* ^[101]^	Retrospective	*N* = 13, TVL (STC) *N* = 16, CRAO/BRAO (STC) *N* = 9, CRAO (IVT)	IVT (rtPA) or STC	No VA outcomes; functional improvement in IVT compared to conservative treatment groups (modified Rankin scale, *P* = 0.006)
Mehboob MA *et al* ^[106]^	Clinical Trial	*N* = 7, Branch/HemiRAO (Conservative) *N* = 7, Branch/HemiRAO (Nd:YAG < 6 hr)	Nd:YAG laser or conservative ${\;}^{a}$	Clinically significant VA improvement in 85.7% of Nd:YAG eyes versus 42.8% of conservative eyes
	
	
Ref, reference; N, sample size; CRAO, central retinal artery occlusion; IAT, intra-arterial thrombolysis; DSA, digital subtraction angiography; VA, visual acuity; logMAR, logarithm of the minimum angle of resolution; RAO, retinal artery occlusion; Sx, symptoms; tPA, tissue plasminogen activator; IVT, intravenous thrombolysis; rtPA, recombinant tissue plasminogen activator; TVL, transient vision loss; STC, standard of care; BRAO, branch retinal artery occlusion; Nd:YAG, neodymium-doped yttrium aluminum garnet. ${\;}^{a}$ Conservative treatment consisted of isovolemic hemodilution, ocular massage, topical beta-blockers, and/or IV acetazolamide				

**Table 2:** Included meta-analyses of thrombolytic studies.


**Author ${\;}^{Ref}$ **	**Inclusion**	***N*** **, Cohort**	**Intervention**	**Visual acuity outcomes**
Hu H *et al* ^[102]^	Studies assessing efficiency of IAT in patients with CRAO compared with standard therapy	*N* = 219, CRAO (IAT) *N* = 240, CRAO (No IAT)	IAT (no specific agent)	VA improvement with IAT: OR 1.52 (*P* < 0.001) VA improved more readily when IAT was administered within 6 hours of symptom onset Urokinase associated with VA improvement, Alteplase was not associated with VA improvement
Huang L *et al* ^[103]^	English studies with ≥ 5 patients with CRAO and conducted IAT treatment and reported VA before and after treatment through Nov 2021	*N* = 507, CRAO (IAT) *N* = 296, CRAO (No IAT)	IAT (streptokinase, urokinase, or alteplase)	SMD in IAT patients: Improved 0.70 logMAR (*P* < 0.0001) VA improvement rate increased in IAT (56%) versus non-IAT (32%, OR 3.55, *P* = 0.0005) IAT < 6 h:r OR 4.60, *P* = 0.02 IAT > 6 hr: OR 3.36, *P* = 0.005
Wang X *et al* ^[104]^	English studies with ≥ 5 patients reporting VA outcomes after CRAO treated with thrombolysis (excluded case reports, BRAO, RVO) through March 2019	*N* = 121	IVT (rtPA)	VA improvement in 40.4% of patients treated with IVT versus 13% treated with conservative management
Huang L *et al* ^[105]^	English studies with ≥ 5 patients with CRAO and conducted IVT treatment with alteplase and reported rate of VA improvement or compared VA to control group	*N* = 157, CRAO (IVT) *N* = 159, CRAO (No IVT)	IVT (alteplase)	IVT versus no IVT: Rate of best VA improvement: OR 5.97 (95% CI 2.77 to 12.86, statistically significant) Rate of final VA improvement: OR 5.25 (95% CI 2.45 to 11.24, statistically significant) Final VA absolute improvement: MD –0.10 (95% CI –0.32 to 0.12, not significant)
Mac Grory B *et al* ^[97]^	Studies with ≥ 5 patients with RAO evaluating IVT with sufficient detail (patient-level details) concerning VA outcomes	*N* = 238	IVT (alteplase, streptokinase, urokinase)	15.2% VA recovery ${\;}^{a}$ with IVT alteplase between 4.6 and 6 hours (*P* = 0.03) 17.7% VA recovery with IVT within 4.5 hours (*P* = 0.0005) Time to treatment was associated with recovery rate (*P* = 0.01) and final visual acuity (*P* = 0.002)
	
	
Ref, reference; N, sample size; IAT, intra-arterial thrombolysis; CRAO, central retinal artery occlusion; VA, visual acuity; OR, odds ratio; logMAR, logarithm of the minimum angle of resolution; BRAO, branch retinal artery occlusion; RVO, retinal vein occlusion; IVT, intravenous thrombolysis; rtPA, recombinant tissue plasminogen activator; MD, mean difference; CI, confidence interval ${\;}^{a}$ VA recovery was defined as functional recovery of vision with a final visual acuity of 20/100 or better				

**Table 3:** Included studies of hyperbaric oxygen for retinal artery occlusion.


**Author ${\;}^{Ref}$ **	**Study type**	***N*** **, Cohort**	**Intervention**	**Visual acuity outcomes**
Rozenberg A *et al* ^[109]^	Retrospective	*N* = 121, RAO (HBOT with STC) *N* = 23, RAO (STC)	3 HBOT sessions 8 hours apart, 90 minutes each (1 ${\;}^{st}$ session 2.4 ATA and rest at 2 ATA), then HBOT once daily until no improvement in VA	HBOT: VA improvement from 2.89 to 2.15 logMAR (*P* < 0.001) Conventional: VA improvement from 3.04 to 2.80 logMAR (*P* = 0.24)
Rosignoli L *et al* ^[92]^	Retrospective	*N* = 15, CRAO (HBOT) *N* = 33, CRAO (No HBOT)	HBOT based on US Navy diving manual, discontinued if no improvement in VA during initial treatment	HBOT: VA improvement from 2.44 to 2.34 logMAR (*P* = 0.14) Conventional: VA improvement from 2.29 to 2.00 logMAR (*P* = 0.10)
Schmidt I *et al* ^[110]^	Retrospective	*N* = 14, NA-BRAO (Hemodilution) *N* = 14, NA-BRAO (HBOT) ${\;}^{a}$	Marx protocol – presenting sessions of 10 min compression, followed by a hyperbaric phase of 90 min (2.4 atm) and 15 min of decompression - Aimed to perform HBOT 5 × within 48 hours, with 3 treatments within the first 24 hours - Additional HBOT was performed when VA was improved but reperfusion was not secured	HBOT: VA improvement from 0.18 to 0.69 decimals (*P* < 0.0001) Control: VA improvement from 0.23 to 0.32 decimals (*P* < 0.0009) – Significant difference in final VA between groups (*P* < 0.0009)
Chiabo J *et al* ^[112]^	Prospective	*N* = 19, CRAO *N* = 12, BRAO	2 daily HBOT sessions (max 2.5 atm, 90 minutes) until revascularization was observed	VA improvement by ≥ 0.3 logMAR at 1 month for 15/31 eyes VA improvement from 1.5 to 1.1 logMAR ${\;}^{b}$
Lopes AS *et al* ^[111]^	Retrospective	*N* = 13, RAO	Phase 1: 2 daily sessions of 100% O2 at 2.5 atm for 90 minutes for 3 consecutive days Phase 2: 1 session per day if VA improved and until it was stabilized	VA improvement from 2.3 to 0.7 logMAR (*P* = 0.007)
Masters TC *et al* ^[108]^	Retrospective	*N* = 39, CRAO	Initial treatment was 2.8 ATA for 90 minutes followed by staged decompression After, HBOT 2 × daily at 2.4 ATA for 90 minutes with 2 air breaks for 9 additional treatments	VA improvement in 72% of patients Mean 5.05 modified Snellen lines of improvement ${\;}^{b}$ – Treatment < 12 of sx onset: 6.11 mean lines of improvement
Rosignoli L *et al* ^[92]^	Meta Analysis ${\;}^{c}$	*N* = 207, CRAO (HBOT) *N* = 89, CRAO (No HBOT)	Any HBOT treatment protocol	No significant improvement in final VA compared to control (*P* = 0.83) No significant differences in change in VA from initial to final presentation (*P* = 0.52) Heterogeneity test: *P* < 0.1
	
	
Ref, reference; N, sample size; RAO, retinal artery occlusion; HBOT, hyperbaric oxygen treatment; STC, standard of care; ATA, atmosphere absolution; VA, visual acuity; logMAR, logarithm of the minimum angle of resolution; CRAO, central retinal artery occlusion; NA-BRAO, non-arteritic branch retinal artery occlusion; atm, atmospheres; ${\;}^{a}$ HBOT initiated in patients where the situation was more serious and the need for any therapeutic success was greater, since the BRAO had affected the functional better eye; ${\;}^{b}$ No statistical analysis reported for VA changes; ${\;}^{c}$ English studies with CRAO patients treated with HBOT and primary outcome assessment with initial and final visual acuity, excluding case reports, reviews, and noncomparative data				

**Table 4:** Included studies of other treatments for retinal artery occlusion.


**Author ${\;}^{Ref}$ **	**Study type**	***N*** **, Cohort**	**Intervention**	**Visual acuity outcomes**
Ikeda HO *et al* ^[117]^	Clinical Trial	*N* = 3, NA-CRAO (Sx 3 to 48 hour; 25 µg) *N* = 6, NA-CRAO (Sx 3 to 48 hour; 50 µg)	Daily intravitreal injections of novel neuroprotectant KUS121 (Kyoto University Substance 121) for 3 days	Low Dose: VA improvement from 2.46 ± 076 to 1.3 ± 2.3 ETDRS letters ${\;}^{a}$ High Dose: VA improvement from 1.97 ± 0.62 to 3.7 ± 3.5 ETDRS letters ${\;}^{a}$
Suzuki T *et al* ^[113]^	Prospective	*N* = 21, CRAO	10 µg/day intravenous liposomal PGE1 1 × /day for 7 days – If VA improved, treatment extended for 14 days	VA improvement from 2.18 to 1.54 logMAR at 1 month (*P* < 0.05) and 1.53 logMAR at 3 months (*P* < 0.05)
Malbin B *et al* ^[114]^	Case Series	*N* = 6, CRAO	Twice daily IV infusion 40 μ g PGE1 until vision stabilized for 24 hours	VA improvement from 2.73 to 1.48 logMAR (*P* = 0.025)
Shah R *et al* ^[120]^	Retrospective	*N* = 297, NA-CRAO ( < 30 days, conservative) *N* = 116, NA-CRAO ( < 30 days, no treatment)	Conservative treatment: anterior chamber paracentesis, ocular massage, IOP lowering drugs, paper bag inhalation	Conservative: 108 (93.1%) with VA < 20/100 at follow up No treatment: 274 (92.3%) with a VA of < 20/100 at follow-up No significant difference in VA outcomes between groups (*P* = 0.8)
Cisiecki S *et al* ^[119]^	Case Series	*N* = 6, RAO ( < 24 hour, vitrectomy with arteriotomy) *N* = 6, RAO ( < 24 hour, vitrectomy with arteriotomy and neurotomy)	Step 1: Conservative treatment with IOP-lowering drugs, ocular massage, and paracentesis Step 2: Vitrectomy – Single embolus: arteriotomy – Unidentifiable embolus or multiple emboli/low VA: arteriotomy and neurotomy	Mean VA improvement from 1.94 to 2.04 logMAR ${\;}^{a}$ – No significant improvement from baseline to final VA in each group – Difference in final VA between groups (arteriotomy: 1.65 logMAR, arteriotomy and neurotomy: 2.45 logMAR, *P* = 0.038)
	
	
Ref, reference; N, sample size; NA-CRAO, non-arteritic central retinal artery occlusion; Sx, symptoms; VA, visual acuity; ETDRS, early treatment diabetic retinopathy study; PGE1, prostaglandin E1; logMAR, logarithm of the minimum angle of resolution; IV, intravenous; IOP, intraocular pressure ${\;}^{a}$ No statistical analysis performed				

### Management and Treatment

Treatment options for the acute management of RAO varies depending on the etiology of occlusion. However, no matter the presentation or etiology, it is critical for patients with RAO to be referred to an emergency department at a stroke center for urgent work up.^[1,22]^ Among all patients, development of RAO is an important indicator of an underlying disease process that may imminently cause more life-threatening sequelae.

For patients who present with sudden onset, monocular vision loss and symptoms of GCA (new headache, jaw claudication, unexplained fever, signs or symptoms of vascular abnormalities), immediate workup, and treatment with high dose of glucocorticoids should be administered promptly.^[76,77]^ Although the visual loss is most likely to be irreversible,^[78,79,80]^quick administration of glucocorticoid therapy is important to prevent future ischemic events and other sequelae of the disease.^[81]^

In the case of patients with non-arteritic RAO, a full cardiac and stroke work up is recommended by the American Academy of Ophthalmology, the American Heart Association, and the American Stroke Association due to the risk of concurrent stroke and other cerebrovascular diseases observed in multiple studies.^[1,22]^ In a retrospective, population-based study in Taiwan, the rate of stroke in the three years following RAO development was calculated and compared to a control population.^[82]^ When adjusted for age, sex, and selected comorbid disorders, the hazard ratio of stroke development for RAO patients was 2.07 times higher than that of controls and 3.34 times higher in the ≤ 60-year-old subgroup.^[82]^ In 2023, Wai et al published a large-scale TriNetX study of 34,874 patients with RAO and the rate of death, stroke, and myocardial infarction at various intervals following RAO presentation.^[83]^ Overall, these patients demonstrated a significantly increased risk of all three conditions at both short- and long-term follow up.^[83]^ Multiple other studies have also demonstrated increased risk of stroke, transient ischemic attack (TIA) and amaurosis fugax in the year before and after RAO development.^[84,85,86,87]^ Additionally, studies of patients who received diffusion-weighted MRI of their brain following RAO still demonstrated silent brain infarctions and diffuse white matter abnormalities in the absence of other neurological symptoms.^[68,88]^ These findings were often not identifiable on computed tomography (CT) scan.

While data on RAO and stroke co-occurrence has been a well-known phenomenon, prompt evaluation and thorough work up of patients who present with CRAO is still largely insufficient.^[89]^ In a study utilizing the Swiss Stroke Registry, among 397 patients who presented with CRAO, 25.6% arrived at the hospital within 4 hours of symptom onset and had a lower rate of emergency referrals compared to patients presenting with ischemic stroke symptoms.^[90]^ Symptom-to-door time was significantly longer among patients with CRAO compared to stroke at 852 minutes versus 300 minutes. In a study of 181 CRAO patients in Atlanta, Georgia, only 34% presented within 24 hours of vision loss, with these patients being more likely to be admitted to the hospital and receive comprehensive stroke work up compared to those who presented after 24 hours since symptom onset.^[91]^

Even amongst patients who present to the emergency department, thorough workup is not often performed. In a study by Yousuf et al, the United States National Emergency Department Sample Database was checked for patients with a primary diagnosis of RAO presenting to the emergency department between 2006 and 2014.^[92]^ Among the 2802 RAO patients, 20.3%, 7.1%, and 23.8% received a form of brain imaging, carotid imaging, or cardiac testing, respectively, with only 4.1% of patients receiving testing in each of these three categories. Further, only half were hospitalized after presentation. An important limitation of this study, though, is that it did not include workups performed as an outpatient, only those performed by the emergency department or inpatient were included, indicating that the study may be under-representing the true rate of stroke work up for these patients.

Nevertheless, treatment for acute presentation of RAO is still debated and without united protocol.^[93]^ In a study by Lee et al, among 91 patients diagnosed with RAO at a single institution, half received no acute treatment for CRAO apart from antiplatelet/anticoagulation pharmacotherapy.^[89]^ Various therapies have been studied for many years, including thrombolytic treatments as well as more conservative options, however, most of these studies have been largely limited by low population size, long symptom-to-treatment time, and poorly defined primary visual outcomes. Among clinicians, no one treatment is preferred, although many still treat with t-PA after a discussion of the risks and benefits.

### Recent Developments in the Acute Treatment of Retinal Artery Occlusion

In the current systematic review, of the 606 unique articles identified on PubMed and Scopus between the publishing years of 2019 and 2023, 25 unique articles met inclusion criteria for the current systematic review in accordance with the Preferred Reporting Items for Systematic Reviews and Meta-Analyses (PRISMA) guidelines [Figure 1]. Two of the articles included both a retrospective cohort study and a meta-analysis and were thus included as four separate studies making a total of twenty-seven studies.^[94,95]^ Fourteen of these studies were retrospective, five were prospective studies, two were clinical trials, and six were meta-analyses. Types of therapies included medical or laser thrombolysis, hyperbaric oxygen therapy, intravenous prostaglandin E1, conservative therapy only, novel neuroprotectant, electrical stimulation, varied supplements, and vitrectomy with arteriotomy and neurotomy.

#### Thrombolysis

Among those reports describing thrombolysis, four studied the use of intra-arterial thrombolysis (IAT), five used intravenous thrombolysis (IVT), and one utilized a method of Neodymium-Doped Yttrium Aluminum Garnet (Nd:YAG) laser embolysis [Table 1]. Across the four studies that investigated the use of IAT, two reported on the use of urokinase and the other two reported on the use of tPA. Overall, all four reported quantitative improvement in the VA by logarithm of the minimum angle of resolution (logMAR) with two demonstrating a statistically significant improvement in VA following treatment.^[96,97]^ The study by Ko et al also demonstrated a statistically significant improvement in VA one month after IAT, however, this improvement became insignificant by last visit.^[98]^ The last study by Kim et al only reported a VA improvement in 10% of patients included.^[99]^

Five studies also investigated the use of IVT (with either tPA or an unspecified drug), with three demonstrating statistically significant improvements in VA following treatment,^[100,101,102]^ and one demonstrating a trend toward improvement for patients treated with IVT compared to conservative treatment, however, this was not statistically analyzed.^[103]^ A study by Schönecker et al also investigated the use of IVT for CRAO with the primary outcome of function improvement according to the modified Rankin Scale.^[104]^ In this article, those treated with IVT demonstrated a statistically significant improvement.

In total, these nine studies included 386 patients treated with either IAT or IVT. Six of these nine thrombolytic studies included information about adverse events following treatment.^[97][98][100,101,102][104]^ While rates of risk of serious adverse events, including intracranial hemorrhage, was low across all studies, nine patients were found to have a bleeding event,^[97,98,100,101]^ with one fatal episode of intracranial hemorrhage in a patient with a “do not resuscitate” order.^[98]^ Additionally, most of these studies only investigated use of thrombolysis for patients with CRAO as opposed to BRAO. This is an important distinction as BRAO can sometimes present with visible emboli or thrombus from calcium or Hollenhorst plaque, neither of which would be susceptible to pharmacologic thrombolysis.

Five meta-analyses that investigated the use of thrombolytics were also identified for inclusion in the current review. Two were limited to IAT with either alteplase, streptokinase, or urokinase.^[105,106]^ Both demonstrated increased odds ratio of visual improvement among patients treated with IAT, with decreasing rates when treatment was implemented 6 hours after symptom onset [Table 2]. Both studies also demonstrated multiple bleeding events or ischemic stroke events among patients treated with IAT.

Three meta-analyses reported on studies treating RAO with IVT with recombinant tPA, alteplase, streptokinase, or urokinase.^[94,107,108]^ Similar to the IAT studies, all three demonstrated increased rates of visual improvement or absolute visual improvement [Table 2], although the 2022 study by Huang et al demonstrated no significant difference in VA change at final follow up between those treated with IVT versus those who did not.^[108]^ Additionally, the 2020 study by Mac Grory et al demonstrated that time to treatment was associated with greater recovery rate and final VA.^[94]^ Again, similar to the IAT studies, all three meta-analyses also reported bleeding events among those treated with IAT as well as neovascularization and recurrent retinal ischemic events [Table 2].

Overall, although some studies appear to demonstrate promising visual results for patients treated with t-PA, larger-scale, randomized controlled trials still must be performed to further validate these results. Current studies and trials have often been limited by variability, but mostly low population size, long symptom-to-treatment time, and poorly defined primary visual outcomes, indicating a need for a larger study to more strongly suggest that the potential visual benefits of thrombolysis may outweigh the risk of bleeding. Given the stroke-like etiology of RAO, it is also reasonable that a 4.5-hour window may be considered the preferred timeline in future studies to receive thrombolysis to accurately assess vision-saving effects and prevent collateral damage similar to the protocol in ischemic strokes. Although the exact length of time to irreversible retinal damage is still debated, as previously mentioned, time to treatment will undoubtedly play an important role in the success of thrombolysis in visual outcomes. Nevertheless, among clinicians today, no one treatment is actively preferred or recommended, however, many still treat with t-PA after a discussion of the risks and benefits.

Another small trial was also included in the current review which detailed the use of laser embolysis for RAO. The 2021 study investigated the efficacy of the addition of Nd:YAG laser embolysis for patients with BRAO or hemi-RAO compared to conventional treatment alone in a trial of 14 patients.^[109]^ In this study, seven patients were treated with both conventional treatment and Nd:YAG embolysis within 6 hours of symptom onset, with 6/7 eyes demonstrating clinically significant VA improvement (defined as improving to 20/200 or better) compared to 3/7 eyes in the group treated with conventional options alone. Notably, five of the seven patients treated with laser embolysis did develop vitreous hemorrhage, although these were all noted to be stable by three-month follow up.

#### Hyperbaric Oxygen Therapy

Six articles included in the current review studied the use of hyperbaric oxygen treatment (HBOT) for RAO, a therapy that has been proposed and investigated in various retinal disease. It has been theorized that HBOT may be a useful therapy for ischemic retinal diseases because of the diffusion of oxygen from the choroidal circulation to the retina.^[110]^ However, it has also been proposed that increased oxygen delivery to the retina may lead to subsequent autoregulated vasoconstriction of the retinal vessels, leading to decreased oxygen delivery.^[111]^ In the current review, five of the included studies were small, retrospective studies with a range of 13 to 121 patients treated with HBOT of varying duration [Table 3]. Although there was large variation in methods and treatment duration across these studies, three reports demonstrated statistically significant improvement in VA among patients treated with HBOT;^[112,113,114]^ one of them also showed statistically significant improvement in patients solely treated with conventional standard of care.^[113]^ The 2023 study by Chiabo et al also observed clinically important improvement in 15 of 31 eyes treated with HBOT and a trend of improvement in mean VA, however, neither of these outcomes were statistically analyzed.^[115]^

One article did not observe any improvement in VA in either HBOT or control groups, although this treatment regimen did specify that treatment was to be discontinued if no improvement was seen in the initial treatment.^[95]^

One meta-analysis was also included in the current study which investigated studies comparing HBOT therapy to no oxygen therapy among patients with RAO. This study showed no significant improvement in final VA compared to the control group (*P* = 0.83) nor in change in VA from initial to final presentation (*P* = 0.52). However, this meta-analysis also included a heterogeneity test with *P* < 0.1 indicating significant heterogeneity between included studies.

#### Other Treatments

The remaining five articles included in the current review studied the use of more conservative or novel therapeutics for non-arteritic RAO [Table 4]. Two studies reported on the use of intravenous prostaglandin E1 for CRAO,^[116,117]^ which has been hypothesized to be helpful in the vasodilation of retinal vessels thus increasing delivery of oxygen to the retinal tissues.^[118]^ Additionally, some have also theorized that anti-platelet aggregation properties of prostaglandin E1 may also decrease any thrombosis of the vasculature.^[119]^ The first study utilized 10 µg/day of intravenous liposomal prostaglandin E1 for seven days among 21 patients with extension of treatment to 14 days if VA improved by day seven,^[116]^ and the second investigated six patients who had been treated with two daily IV infusions of 40 µg prostaglandin E1 until vision had stabilized for at least 24 hours.^[117]^ Although both studies lacked a control group for comparison, they did both demonstrate a statistically significant improvement in VA following treatment without reported adverse events.

A study by Ikeda et al enrolled nine patients in a phase I/II clinical trial at Kyoto University to investigate the safety of the novel neuroprotectant Kyoto University Substance 121 (KUS121) for three days in patients with non-arteritic CRAO with symptoms presenting for 3–48 hours.^[120]^ In this trial, three patients received daily 25 µg intravitreal injections of KUS121 for three days and six received 50 µg intravitreal injections of KUS121 for three days. By the 12-week follow up, all nine patients demonstrated improvement in VA without serious adverse events. Mild adverse events known to occur with intravitreal injections did occur, with all nine patients experiencing immediate increase in intraocular pressure following injection, although pressure did universally decrease after paracentesis. The patients who received low-dose KUS121 also developed episodes of macular edema with cystoid spaces and iris neovascularization (NVI), and high-dose patients demonstrated some instances of foveal retinal detachment, macular edema with cystoid spaces, and recurrent retinal ischemia, although all adverse events were resolved either without treatment or with panretinal photocoagulation for the NVI cases.

A phase II clinical trial is currently recruiting to further investigate the safety and efficacy of intravitreal KUS121 for acute, non-arteritic CRAO.^[121]^ The study plans to randomize 75 subjects to high dose, low dose, or sham to receive KUS121 injections between 3–48 hours of onset of CRAO. Primary endpoint will be proportion of participants who gain 15 letters or more in BCVA at 12 weeks compared to baseline. Safety evaluations will continue for one year after injection.

A case series by Cislecki et al reported the use of vitrectomy with arteriotomy alone or with arteriotomy and neurotomy among 12 patients who presented with RAO more than 24 hours after symptom onset.^[122]^ These patients were initially treated conservatively and when no improvement was made, those with a single embolus identified (six patients) underwent vitrectomy with arteriotomy while those with unidentifiable embolus or multiple emboli underwent vitrectomy with arteriotomy and neurotomy. These patients were followed for 12 months with no significant improvement from baseline to final VA in each group, but with a significant difference in final VA between those treated with arteriotomy alone (1.65 logMAR) versus those treated with arteriotomy and neurotomy (2.45 logMAR, *P* = 0.038).

The final study included in the current review was a retrospective review of conservative treatments for RAO, including anterior chamber paracentesis, ocular massage, intraocular pressure lowering drugs, and paper bag inhalation.^[123]^ Four hundred forty-one patients were included who were diagnosed with non-arteritic CRAO within 30 days of symptom onset and an initial VA of 20/200 or worse and at least 90 days of follow up. Of these, 297 patients were treated with conservative treatments, and 116 were provided no therapies. Among those treated with conservative therapies, 93.1% showed VA improvement to better than 20/100 compared to 92.3% of those without treatment, which did not reveal any statistically significantly difference.

#### Subacute management 

Subacute management of RAO mainly focuses on the treatment of sequelae of retinal ischemia as well as secondary prevention of recurrence or stroke. One commonly seen complication of RAO is NVI and subsequent neovascular glaucoma in the months after symptom onset which may then be treated with intravitreal anti-VEGF injections.^[124,125,126]^ A study by Jung et al reported an incidence rate of 10.9% of NVI and 6.4% of neovascular glaucoma in the months following CRAO.^[127]^ Although severely narrowed carotid arteries were observed in 3 of 12 patients with NVI, the remaining patients had no other predisposing conditions for NVI. Another study by Lo et al, reported on additional risk factors for the development of neovascularization following CRAO and found that chronic kidney disease and glaucoma history had a hazard ratio of 9.27 and 7.52, respectively.^[128]^

Secondary prevention of RAO is also critically important to prevent further occlusive or inflammatory events from the underlying, concurrent disease. Diagnosis of concurrent diseases may sometimes require a multidisciplinary approach as does treatment. As mentioned previously, GCA is a key differential for RAO and must be thoroughly investigated with appropriate laboratory, imaging, and biopsy studies in order to be included or excluded as etiology of retinal ischemia. As discussed previously, among patients with a cardiac or atheroembolic origin of emboli, thorough cardiac (i.e., electrocardiography and echocardiography), carotid imaging (i.e., carotid Doppler, computed tomography angiography [CTA], magnetic resonance angiography [MRA]), and neurological work up (i.e., MRI and/or CT of brain) should be performed.^[1,22]^ Stringent antiplatelet and anticoagulant therapy must also be started along with control of risk factors such as smoking, hypertension, hyperlipidemia, obesity, valvular disease, and arrhythmia, similar to patients who have had a stroke.^[1,22]^ Additionally, for patients with auto-immune or inflammatory origins, proper control with steroids and other indicated treatments should also be started.^[19,22]^

Some investigators are also looking at possible treatments to alleviate some of the chronic damage caused by RAO. One such treatment is transdermal electrical stimulation (TdES). Electrical stimulation has been used in various ocular diseases such as retinitis pigmentosa, glaucoma, and optic neuropathy to improve visual function^[129]^ with some studies hypothesizing that it may be able to do so by activating insulin-like growth factor-1, brain derived neurotrophic factor, ciliary neurotrophic factor, and fibroblast growth factor-2.^[130]^ However, most studies have investigated the use of transcorneal stimulation as opposed to transdermal, with the caveat that transcorneal stimulation may cause corneal epithelial damage. A 2023 phase I clinical trial used TdES among patients with fixed symptoms from RAO for over six months and decimal VA ranging from hand motion to 0.7.^[130]^ Five patients were enrolled and treated with TdES at two-week intervals. By the 12-week follow up, there were no adverse events reported, however, VA improvement in these eyes also varied greatly without any consistent improvement in vision.

A retrospective case series by Fernandez-Vega et al in 2020 also reported on the use of various supplements for a variety of vascular diseases affecting the visual field including more chronic state RAO.^[131]^ The other major diseases included were non-arteritic ischemic optic neuropathy, and homonymous hemianopsia with seven RAO patients included in the study. Supplements provided to patients included active complex Q10 gold, B vitamins, citicoline, Visan capsules, and aspirin. Although statistical analysis was not provided for visual outcomes, the authors did report an improvement in visual field index among patients with RAO.

##  SUMMARY

Overall, CRAO should be considered an ophthalmic emergency by clinicians and should be evaluated quickly following symptom onset with a prompt referral to an emergency department with a stroke center. Due to the various etiologies of CRAO, including both arteritic and non-arteritic causes, a thorough cardiovascular work up must be performed including for vasculitidies such as GCA in order to prevent further cardiovascular events. It is thus vitally important for both optometrists, ophthalmologists, and general practitioners to be able to recognize the signs and symptoms of RAO in order to be able to adequately triage these patients and appropriately refer them to stroke centers for work up for concurrent events or risk factors for imminent stroke.

Although there have been many studies regarding the best method of therapy for these patients, there is still no current gold-standard treatment in the acute or chronic phase for them, and many are left with significant visual field loss after occlusion. However, there have been many promising treatments that have been studied recently and are undergoing clinical trial including intravenous and IAT, HBOT, neuroprotection, prostaglandin administration, and even surgical or laser options. These modalities still must undergo large-scale clinical trials to definitively prove significant ophthalmic benefit.

##  Financial Support and Sponsorship

None.

##  Conflicts of Interest

None.
